# An Atypical Presentation of Enteroviral Meningitis in an Immunocompetent Adult Man

**DOI:** 10.7759/cureus.96231

**Published:** 2025-11-06

**Authors:** Klaudia Greer, Jonathan Brutti, Temiloluwa Kowobari, Lorena Del Pilar Bonilla

**Affiliations:** 1 Herbert Wertheim College of Medicine, Florida International University, Miami, USA; 2 Family Medicine, Baptist Health South Florida, Miami, USA; 3 Translational Medicine, Herbert Wertheim College of Medicine, Florida International University, Miami, USA

**Keywords:** atypical presentation, csf pcr, enterovirus, headache, meningitis

## Abstract

Aseptic meningitis is inflammation of the meninges characterized by cerebrospinal fluid (CSF) pleocytosis and negative bacterial cultures. Viral infections are the most common cause, with enteroviruses (EV) accounting for the majority of cases worldwide. Typical presentations include fever, headache, and meningeal signs; however, atypical cases can occur, leading to diagnostic uncertainty. Early recognition and lumbar puncture (LP) are essential to avoid misdiagnosis. This case highlights an unusual presentation of enteroviral meningitis without fever or meningeal signs in an immunocompetent adult man.

A 40-year-old man with a history of hypertension presented to the emergency department (ED) with a gradual-onset, severe headache, neck pain radiating to the scalp, mild nausea, and transient blurry vision. On examination, he was alert and oriented, afebrile, and in no acute distress. There was bilateral paraspinal tenderness but no nuchal rigidity or meningeal signs. Cardiovascular and neurologic examinations were unremarkable. Laboratory testing revealed mild leukocytosis and elevated liver enzymes. Brain and cervical spine imaging were unremarkable. LP showed clear CSF with an elevated opening pressure (26 mmHg), elevated protein (87 mg/dL), normal glucose (65 mg/dL), and mild pleocytosis. CSF Gram stain and cultures were negative. A meningitis-encephalitis polymerase chain reaction (PCR) panel was positive for EV but negative for bacterial and herpesviral pathogens, while a separate EV PCR was negative. The patient was started empirically on intravenous (IV) ceftriaxone, vancomycin, and acyclovir. Following confirmation of EV, antimicrobials and antivirals were discontinued, and supportive management with fluids, analgesics, and gastrointestinal prophylaxis was initiated. His symptoms resolved within 48 hours, and he was discharged in stable condition.

This case demonstrates that enteroviral meningitis can present without fever, neck stiffness, or other meningeal signs, which may delay diagnosis. Classic meningeal signs have limited sensitivity, and their absence should not exclude meningitis in patients with persistent, severe headache. Additionally, the discordant PCR results emphasize that molecular assays are not infallible; repeat testing should be considered when clinical suspicion remains high. Recognition of atypical presentations is crucial to guide appropriate management. This case underscores that early LP and comprehensive diagnostic evaluation are essential when clinical findings are nonspecific. Maintaining a broad differential and relying on clinical judgment rather than physical signs alone can help prevent missed or delayed diagnoses.

## Introduction

Aseptic meningitis is defined as meningeal inflammation with cerebrospinal fluid (CSF) pleocytosis and negative bacterial cultures [1]. While non-infectious etiologies such as autoimmune disease, malignancy, and drug reactions exist, viruses are the most frequent cause [1]. Common viral causes include enterovirus (EV), herpes simplex virus (HSV), and varicella-zoster virus (VZV) [2]. The illness typically presents with acute-onset headache, fever, and meningeal signs and generally follows a benign course [3]. Viral meningitis shows a seasonal pattern, peaking in summer and autumn in temperate regions [4].

EVs, small non-enveloped RNA viruses of the Picornaviridae family, are the leading cause of viral meningitis worldwide [5]. Epidemiological studies confirm the significant burden of enteroviral meningitis in adults. For instance, in Denmark, EVs accounted for 39% of adult viral meningitis cases between 2015 and 2020 [2]. More than 116 human EV genotypes have been identified, with EV-A71, CVB1-5, E3-7, E9, E11, E18, and E30 most often implicated in meningitis [6]. Transmission occurs primarily via the fecal-oral route, though spread through respiratory secretions and contact with contaminated objects is also possible [6].

Clinically, enteroviral meningitis often presents with fever, headache, and meningeal irritation, though adolescents and adults may also experience malaise, myalgia, photophobia, nausea, and vomiting [2]. CSF findings typically show mild to moderate pleocytosis, mildly to moderately elevated protein, and normal glucose levels [7].

In suspected central nervous system infections, hospitalization is recommended for monitoring, supportive care, and empiric antimicrobial therapy while awaiting culture results [7]. Neuroimaging may be used to exclude mass lesions or abscesses, and acyclovir is administered if HSV infection is suspected due to its high morbidity and mortality [7]. The use of polymerase chain reaction (PCR) has enhanced diagnostic accuracy, enabling rapid confirmation of EV infection [5]. However, assay performance can vary, and false-negative or discordant results may occur. Once EV is identified via PCR, antibiotics can be safely de-escalated. Treatment is mainly supportive, using analgesics, antipyretics, and fluids, and most patients recover within one to two weeks [7].

Although atypical presentations of enteroviral meningitis can complicate diagnosis and management, they are rarely reported in the literature. This case describes an immunocompetent adult man with enteroviral meningitis who presented without fever or neck stiffness and had conflicting PCR results, underscoring the diagnostic challenges posed by atypical cases.

## Case presentation

A 40-year-old man with a history of hypertension presented to the emergency department (ED) at 4:10 a.m. with a complaint of a headache that began gradually at 2:00 p.m. the previous day. He reported persistent neck pain radiating to the scalp, mild nausea, and a transient episode of blurry vision that had since resolved. The patient rated the pain as 9/10 in severity and had taken ibuprofen and acetaminophen the day before, with minimal relief. His last dose was at 9:00 p.m. the night prior to presentation. He denied any prior history of headaches or migraines, “worst headache of his life,” sudden-onset headache, fever, chills, loss of consciousness, imbalance, dizziness, back pain, abdominal pain, vomiting, diarrhea, palpitations, shortness of breath, chest pain, or other associated symptoms. He also denied recent travel or animal exposure. The patient was born in Florida, works as an insurance adjuster, and lives at home with his wife and two children, aged 8 and 13.

Physical examination results

The patient’s vital signs on arrival to the ED were within normal limits, except for an elevated blood pressure of 148/103 mmHg. On physical examination, he was well-appearing, in no acute distress, and alert and oriented to person, place, time, and event. There was bilateral paraspinal muscle tenderness to palpation but no nuchal rigidity or other meningeal signs. Neurologic examination revealed no focal deficits. Cranial nerves were intact, motor strength and sensation were normal and symmetric, and speech, gait, and coordination were unremarkable. The head was normocephalic with normal ear and nasal examinations, intact hearing, and no sinus tenderness. Cardiovascular examination showed a regular rate and rhythm without murmurs or pedal edema. Respiratory effort was normal, and the lungs were clear to auscultation. 

Laboratory results 

A complete blood count (CBC), comprehensive metabolic panel (CMP), lumbar puncture (LP), and blood culture with Gram stain were performed in the ED on hospital day 1. The CBC demonstrated leukocytosis of 11.56 K/µL with neutrophilic predominance (76.7%) and a mildly elevated red blood cell (RBC) count of 5.83 M/µL (Table 1). The CMP revealed elevated glucose at 142 mg/dL, a low blood urea nitrogen-to-creatinine ratio of 11.5, elevated liver enzymes (ALT 119 U/L, AST 48 U/L), and an elevated total bilirubin of 1.2 mg/dL (Table 2). The LP was performed in the lateral decubitus position and showed a mildly elevated opening pressure of 26 cm H₂O. CSF analysis from tubes 2 and 3 revealed a clear appearance, 3-8 RBCs, no xanthochromia, and 4-8 nucleated cells. Tube 1 analysis showed elevated protein at 87 mg/dL and normal glucose at 65 mg/dL (Table 3). Influenza A and B antigens were negative, VDRL-CSF was non-reactive, and COVID-19 serology was negative. An in-house meningitis-encephalitis PCR panel of CSF collected at 6:09 a.m. and reported at 1:25 p.m. was positive for EV and negative for *Escherichia coli*, *Haemophilus influenzae*, *Listeria monocytogenes*, *Neisseria meningitidis*, *Streptococcus agalactiae*, *Cytomegalovirus*, HSV-1 and HSV-2, HHV-6, human parechovirus, VZV, and *Cryptococcus neoformans* (Table 4). Interestingly, a separate enterovirus CSF PCR performed by an external reference laboratory on a sample collected at 6:15 a.m. returned a negative result. Peripheral blood and CSF cultures and Gram stains were also negative.

**Table 1:** Complete blood count on admission WBC: white blood cells, RBC: red blood cells, H: high, L: low.

Parameter	Patient result	Reference range
WBC	11.56 × 10³/µL (H)	3.40-11.00 × 10³/µL
RBC	5.83 × 10⁶/µL (H)	4.00-5.70 × 10⁶/µL
Hemoglobin	17.0 g/dL	13.0-17.2 g/dL
Hematocrit	48.2%	38.0%-50.0%
Platelet count	219 × 10³/µL	130-360 × 10³/µL
% neutrophils, absolute	76.7% (H), 8.86 × 10³/µL (H)	40.0%-70.0%, 0.60-3.10 × 10³/µL
% immature granulocytes	0.6% (H)	0.0%-0.4%
% lymphocytes, absolute	13.7% (L), 1.58 × 10³/µL	17.0%-45.0%, 0.60-3.10 × 10³/µL
% monocytes, absolute	8.4%, 0.97 × 10³/µL	3.0%-12.0%, 0.44-1.00 × 10³/µL
% eosinophils, absolute	0.3%, 0.04 × 10³/µL	0.0%-7.0%, 0.00-0.54 × 10³/µL
% basophils, absolute	0.3%, 0.04 × 10³/µL	0.0%-1.0%, 0.00-0.08 × 10³/µL

**Table 2:** Comprehensive metabolic panel on admission BUN: blood urea nitrogen, ALT: alanine aminotransferase, AST: aspartate aminotransferase, H: high.

Parameter	Patient result	Reference range
Sodium	136 mmol/L	136-145 mmol/L
Potassium	4.0 mmol/L	3.5-5.1 mmol/L
Chloride	104 mmol/L	98-107 mmol/L
CO_2_	23 mmol/L	21-32 mmol/L
Glucose	142 mg/dL (H)	70-126 mg/dL
Creatinine	1.13 mg/dL	0.70-1.30 mg/dL
BUN	13 mg/dL	7-18 mg/dL
ALT	119 U/L (H)	16-65 U/L
AST	48 U/L (H)	8-37 U/L
Alkaline phosphatase	63 U/L	50-136 U/L
Total bilirubin	1.20 mg/dL (H)	0.20-1.00 mg/dL

**Table 3:** Analysis of cerebrospinal fluid (CSF) RBCs: red blood cells, H: high, L: low.

Parameter	Tube 1	Tube 2	Tube 3	Tube 4	Reference range
Appearance	Clear	Clear	Clear	Clear	Clear
Total nucleated cells	9 cells/µL	8 cells/µL	4 cells/µL	1 cells/µL	0-10 cells/µL
RBCs	7 cells/µL (H)	8 cells/µL (H)	3 cells/µL (H)	10 cells/µL (H)	0 cells/µL
Neutrophils	52% (H)	36% (H)	31% (H)	18% (H)	0%-7%
Lymphocytes	46%	60%	61%	61%	28%-96%
Monocytes	2% (L)	4% (L)	8% (L)	21%	16%-56%
Eosinophils	0%	0%	0%	0%	0%-1%
Basophils	0 %	0%	0%	0%	0%
Total protein	87 mg/dL (H)	-	-	-	8-32 mg/dL
Glucose	65 mg/dL	-	-	-	40-70 mg/dL
Xanthochromia	Absent	Absent	Absent	Absent	Absent

**Table 4:** Molecular infectious laboratory results CSF: cerebrospinal fluid, PCR: polymerase chain reaction, HSV: herpes simplex virus, HHV: human herpesvirus, VDRL: Venereal Disease Research Laboratory.

Parameter	Result
HSV-1 DNA	Not detected
HSV-2 DNA	Not detected
Enterovirus CSF PCR	Negative
*Escherichia coli* by K1 CSF PCR	Not detected
*Haemophilus influenzae* CSF PCR	Not detected
*Neisseria meningitidis* CSF PCR	Not detected
*Streptococcus agalactiae* CSF PCR	Not detected
*Cytomegalovirus* CSF PCR	Not detected
*Enterovirus* CSF PCR	Detected
HSV-1 CSF PCR	Not detected
HSV-2 CSF PCR	Not detected
HHV-6 CSF PCR	Not detected
Human parechovirus CSF PCR	Not detected
Varicella-zoster virus CSF PCR	Not detected
*Cryptococcus neoformans*/*gattii *CSF PCR	Not detected
VDRL-CSF	Non-reactive

Imaging

**Figure 1:**
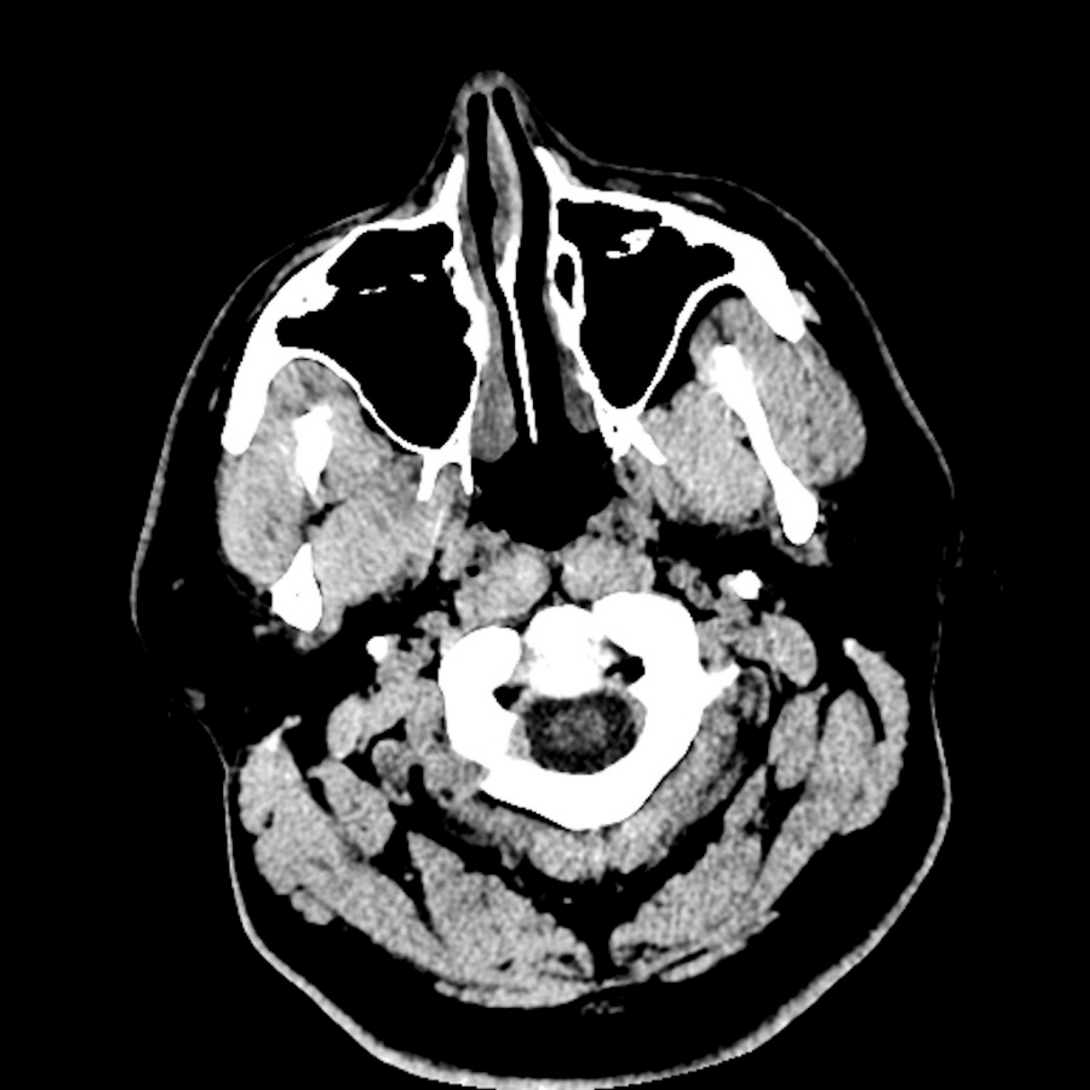
Normal non-contrast computed tomography of the brain No acute intracranial hemorrhage, midline shift, or mass effect.

**Figure 2:**
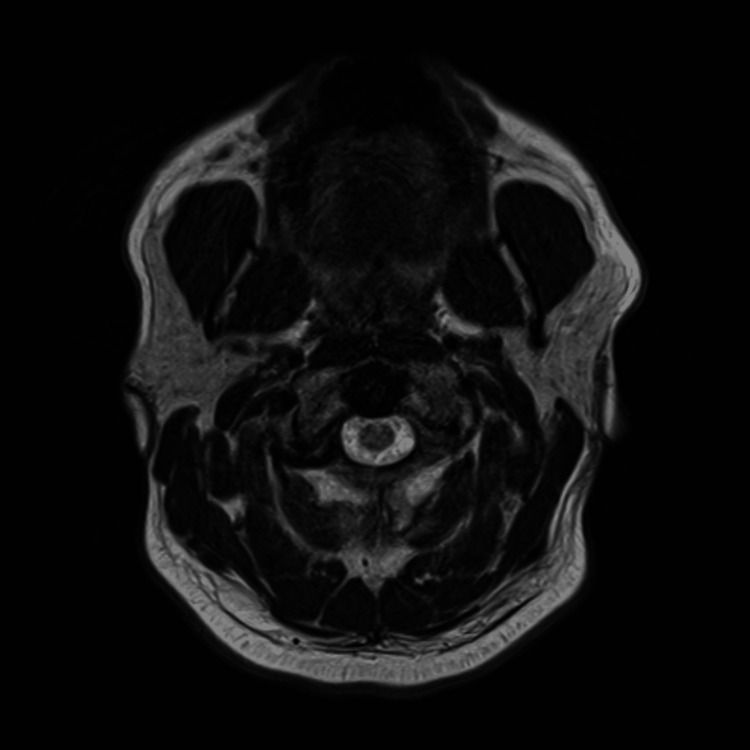
Normal magnetic resonance imaging of the brain with and without contrast No acute intracranial abnormality detected on this motion-degraded examination.

Non-contrast CT scans of the brain and cervical spine performed at 4:53 a.m. were unremarkable (Figure 1). The patient was admitted at 8:25 a.m. for evaluation of an intractable headache. Subsequently, brain magnetic resonance imaging (MRI) with and without contrast, performed at 12:39 p.m., revealed no abnormalities. Both head and neck magnetic resonance angiograms (MRAs) without contrast were also normal (Figure 2).

Treatment and hospital course

While in the ED, the patient received a “migraine cocktail” consisting of a 5 mg intravenous (IV) push of prochlorperazine, a 12.5 mg IV push of diphenhydramine, and a 15 mg IV push of ketorolac. He was also given IV fluids, a 2 g IV piggyback of ceftriaxone, and an 8 mg IV push of dexamethasone. After admission, he received a 1,500 mg IV piggyback of vancomycin and continued IV fluids. Following the positive enterovirus result, he was administered three doses of 900 mg IV piggyback of acyclovir every eight hours. On hospital day 2, an infectious disease consultant recommended discontinuing acyclovir, noting that treatment for enteroviral meningitis is supportive. The patient was also started on 40 mg IV pushes of pantoprazole for gastrointestinal prophylaxis. He continued to receive IV fluids, along with 15 mg IV pushes of ketorolac and 3 mg IV pushes of morphine as needed for pain management.

The patient was hospitalized for a total of two nights for evaluation and management of intractable headache. He was diagnosed with viral meningitis secondary to enterovirus infection and treated supportively. The infectious disease team was consulted, and the patient was expected to make a full recovery with conservative management. After more than 24 hours of observation, he remained hemodynamically stable, afebrile, and reported complete resolution of his headache. Neurologic examination remained normal throughout his hospital stay. The patient was discharged on hospital day 3 with instructions to follow up with his primary care physician within seven days.

## Discussion

Classically, meningitis presents with fever, headache, and neck stiffness [2]. However, in a study of 297 adults with suspected meningitis, the sensitivities of nuchal rigidity, Kernig, and Brudzinski signs were low, approximately 30%, 5%, and 5%, respectively [8]. A meta-analysis of physical examination tests for meningitis also found that Kernig and Brudzinski signs had poor sensitivity, while nuchal rigidity demonstrated only 40%-60% sensitivity [9]. Clinical series further report that only about two-thirds of adults with confirmed meningitis exhibit neck stiffness [10], and roughly one-third of those with viral meningitis lack documented nuchal rigidity on presentation [10]. Rare reports even describe fulminant bacterial meningitis without any meningeal signs, leading to diagnostic delay [11]. Moreover, a population-based study from 2015 to 2019 reported that fever was present in only 48% of adult patients with enteroviral meningitis at admission [12]. This patient’s lack of fever and nuchal rigidity illustrates that a high index of suspicion is warranted in cases of persistent, severe headache with CSF pleocytosis, even when vital signs and physical findings are unremarkable.

Literature on atypical presentations of enteroviral meningitis remains limited, though VZV meningitis is a well-documented example of an infection that can defy classical expectations. VZV meningitis may occur without rash or fever, sometimes presenting as an isolated headache in immunocompetent adults [13]. In such cases, diagnosis relies on CSF PCR testing [13]. Reviews estimate that 33%-60% of VZV meningitis cases lack a vesicular rash, with some patients also afebrile and without neck stiffness [14]. Clinical improvement with acyclovir is typically observed when VZV is the cause [13]. These observations reinforce the importance of early LP and molecular testing when patients present with severe or persistent headache without another clear etiology.

Typical CSF findings in aseptic meningitis include mild to moderate lymphocytic pleocytosis, normal glucose levels, and normal to slightly elevated protein [15]. Rapid CSF EV PCR testing can shorten hospital stay, reduce ancillary testing, and decrease unnecessary antibiotic exposure [16]. While PCR technology is highly valuable, it is not infallible. During a Swiss outbreak of aseptic meningitis affecting both children and adults, CSF PCR demonstrated 85% sensitivity and yielded results within five hours [17]. However, false negatives can occur due to low viral concentrations in the CSF or inhibitory substances interfering with PCR enzymes [17]. In a large evaluation of the FilmArray ME panel, false-negative EV results were also reported, with an overall sensitivity of about 89% [18]. Therefore, a single negative result should not be considered definitive if clinical suspicion remains high. In our case, one assay was positive while another was negative. Repeat testing may be necessary in practice, and discordant results should always be interpreted in the context of clinical and laboratory findings.

Neuroimaging such as CT and MRI may be used to exclude alternative causes of headache but should not delay empiric therapy for suspected meningitis. Head CT prior to LP is not routinely indicated unless the patient presents with focal neurologic deficits, altered mental status, or a history of seizures [19]. Initial priorities include obtaining blood cultures, performing prompt LP, and initiating empiric therapy [20]. Once enteroviral meningitis is confirmed, dexamethasone, antibiotics, and antiviral therapy can be safely discontinued, as the condition is self-limiting and managed supportively [20]. In this case, the patient’s headache resolved within two days with supportive treatment, and he was discharged without complications.

## Conclusions

This immunocompetent adult with enteroviral meningitis presented atypically without fever or nuchal rigidity, illustrating how reliance on physical examination alone can miss cases of viral meningitis. Clinicians should consider aseptic meningitis even in the absence of fever or neck stiffness when patients present with headache and CSF pleocytosis. Laboratory findings also revealed discordance between two EV CSF PCR assays, underscoring that no diagnostic test is infallible. Negative PCR results do not always exclude viral infection; repeat or alternative testing may be warranted when clinical suspicion remains high. This case reinforces the importance of prioritizing clinical judgment over isolated physical or laboratory findings. Empiric antimicrobial coverage should be initiated when indicated, then de-escalated to supportive care once bacterial and HSV etiologies are excluded and enteroviral infection is confirmed or strongly supported. Overall, this case highlights key diagnostic pitfalls when fever and classic meningeal signs are absent and when relying solely on a single molecular assay, emphasizing the need for early LP and careful interpretation of laboratory results.
